# Theragnostic Efficacy of K18 Response in Alcohol Use Disorder with Clinically Significant Fibrosis Using Gut-Liver Axis

**DOI:** 10.3390/ijms23105852

**Published:** 2022-05-23

**Authors:** Manasa Sagaram, Ranganathan Parthasarathy, Sally L. Condon, Charles F. Closson, Maiying Kong, Melanie L. Schwandt, Loretta L. Jophlin, Wenke Feng, Ashutosh J. Barve, Vatsalya Vatsalya

**Affiliations:** 1 Internal Medicine Residency Program, University of Louisville, Louisville, KY 40202, USA; manasa.sagaram@louisville.edu; 2 Division of Gastroenterology, Hepatology and Nutrition, Department of Medicine, University of Louisville, Louisville, KY 40202, USA; ranganathan.parthasarathy@louisville.edu (R.P.); sally.condon@louisville.edu (S.L.C.); charles.closson@louisville.edu (C.F.C.); loretta.jophlin@louisville.edu (L.L.J.); wenke.feng@louisville.edu (W.F.); ashutosh.barve@louisville.edu (A.J.B.); 3 Department of Bioinformatics and Biostatistics, University of Louisville, Louisville, KY 40202, USA; maiying.kong@louisville.edu; 4 Alcohol Research Center, University of Louisville, Louisville, KY 40202, USA; 5 National Institute on Alcohol Abuse and Alcoholism, Bethesda, MD 20892, USA; melanies@mail.nih.gov; 6 Robley Rex VA Medical Center, Louisville, KY 40206, USA

**Keywords:** early-stage ALD, AUD, fibrosis, IL-8, K18M65

## Abstract

(1) Background: Fibrosis in early-stage alcohol-associated liver disease (ALD) is commonly under-diagnosed in routine clinical practice. This study characterized the liver-injury and cell death response in alcohol use disorder (AUD) patients with ALD who also exhibited fibrosis and assessed the efficacy of standard of care (SOC) treatment in the improvement in liver injury. (2) Methods: Forty-eight heavy-drinking AUD patients aged 21–65 yrs. without clinical manifestations of liver injury were grouped by Fibrosis-4 (FIB-4) score, as negative (Gr.1 < 1.45, *n* = 21) or positive (Gr.2 ≥ 1.45, *n* = 27). Patients received 2-weeks (2 w) inpatient SOC. Data on demographics, drinking patterns, liver-injury, immune markers, and liver cell death (K18s) markers were analyzed at baseline (BL) and after 2 w SOC. (3) Results: Lifetime drinking (LTDH, yrs.) and acute heavy drinking (Heavy Drinking Days Past 90 Days [HDD90]) markers were significantly higher in Gr.2 vs. Gr.1. BL ALT, AST, AST:ALT and K18M65 were considerably higher in Gr.2. Dysregulated gut dysfunction and elevated immune activity were evident in Gr.2 characterized by TNF-α, IL-8 and LPS levels. After SOC, Gr.2 showed improvement in AST, ALT, AST/ALT ratio; and in the K18M65, K18M30 and K18M65/M30 ratio vs. Gr.1. The true positivity of BL IL-8 response to predict the improvement in K18M65 to normal levels among Gr.2 patients against those who did not have improvement after 2 w SOC was very high (AUROC = 0.830, *p* = 0.042). (4) Conclusions: Gut dysfunction, elevated cytokine response and necrotic liver cell death were elevated in AUD patients with early-stage ALD. K18 showed promise as a predictive theragnostic factor to differentiate among the AUD patients with early-stage ALD and baseline fibrosis who had improvement in liver injury against those who did not, by the levels of baseline IL-8.

## 1. Introduction

Chronic heavy consumption of alcohol is the cornerstone of the formation of alcohol associated liver disease (ALD), which can lead to steatosis, hepatitis, cirrhosis, and hepatocellular carcinoma. Notably, only 10–20% of chronic heavy drinkers develop severe forms of ALD such as hepatitis and cirrhosis [1]. It is currently unknown how to predict this subset of patients. New and emerging biomarkers previously studied in severe ALD show promise in discriminating which alcohol use disorder (AUD) patients may develop early-stage asymptomatic ALD with fibrosis, and which of these patients may not improve after treatment.

**Figure 1:**
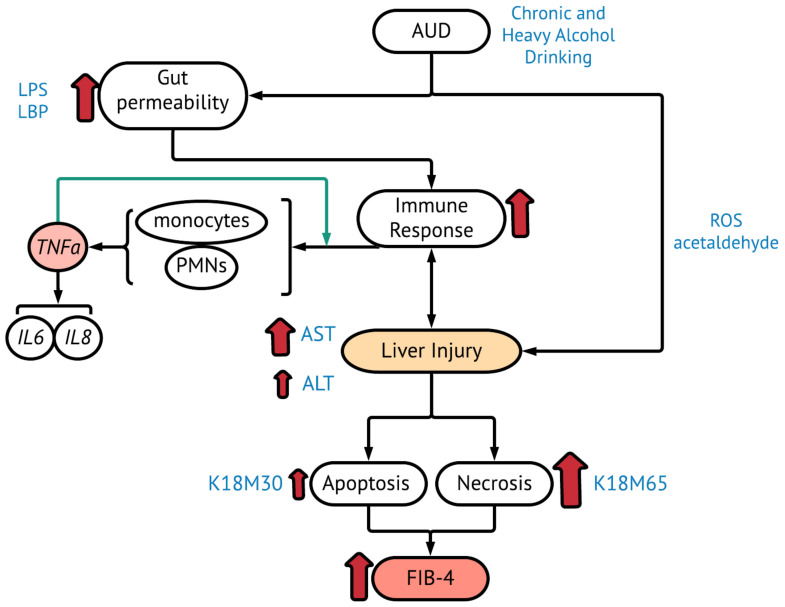
Model to illustrate the mechanism of the development of Early-Stage ALD from AUD; and the mediating inflammatory cytokines, hepatocyte death biomarkers, and liver injury markers.

Elevated gut permeability and disruption of the intestinal epithelial barrier results in an increase in the levels of portal-circulating bacterial endotoxins, such as lipopolysaccharide (LPS), LPS-binding protein (LBP), and soluble cell of differentiation type 14 (sCD14). This leads to the inflammation of the liver and is a well-described pathological pathway of ALD [2]. Resulting liver inflammation leads to the production of tumor necrosis factor-α (TNF-α), among other pro- and anti-inflammatory cytokines, primarily by activated Kupffer cells [3]. Elevated TNF-α, the primary inflammatory mediator, leads to an elevation in several other inducible cytokines, such as interleukins 6, 8, and 1β (IL-6, IL-8, and IL-1β), all of which promote further inflammation, hepatic stellate cell mediated fibrosis, and hepatocyte necrosis (Figure 1) [3]. While these cytokines are loosely associated with ALD, no single biomarker has been identified as a gold standard in predicting the development of ALD with early liver fibrosis in heavy drinkers. This underscores the need for discovery and validation of prognostic non-invasive biomarkers in early-stage ALD.

Cytokeratin 18 (K18) is an intermediate filament protein that is present in simple epithelial tissues and constitutes part of the hepatocyte cytoskeleton [4,5]. Soluble K18 is released from dying cells and can be used in detecting overall cell death (due to apoptosis and necrosis) in the liver [6]. K18 is passively released during necrosis, while during apoptosis K18 is cleaved by caspases [6]. The cleaved form is referred to as K18M30 while the whole protein is referred to as K18M65 [6]. IL-8 expression is regulated by TNF-α [7] and this cytokine response directly contributes to the induction of hepatocyte cell death [8] as we hypothesized in this study (Figure 1). Several studies demonstrate that K18 significantly predicts survival in the late stages of liver disease such as in acute liver failure, viral hepatitis, and NAFLD [9,10,11]. There have been no studies to our knowledge that examine these cytokeratins in AUD patients and their utility to diagnose early-stage ALD [12] with early fibrosis as well as prognosticate the outcome after SOC.

This study evaluated candidate pro-inflammatory cytokines and markers of altered gut-dysregulation as potential diagnostic/prognostic biomarkers in patients with AUD who exhibited early-stage ALD along with a positive fibrosis-4 (FIB-4) score. These biomarkers were also evaluated in the patients over a course of two weeks while receiving inpatient standard of care (SOC). We hypothesized that the candidate biomarkers specific to the necrosis type of the liver cell death, such as K18M65, could be explained by a gut–liver axis response of gut dysregulation and pro-inflammatory cytokines. We also aimed to evaluate the SOC-dependent K18 response that can predict improvement or lack of improvement in early-stage liver injury in patients with fibrosis.

## 2. Results

### 2.1. Demographics, Nutrition and Drinking Profile

**Figure 2:**
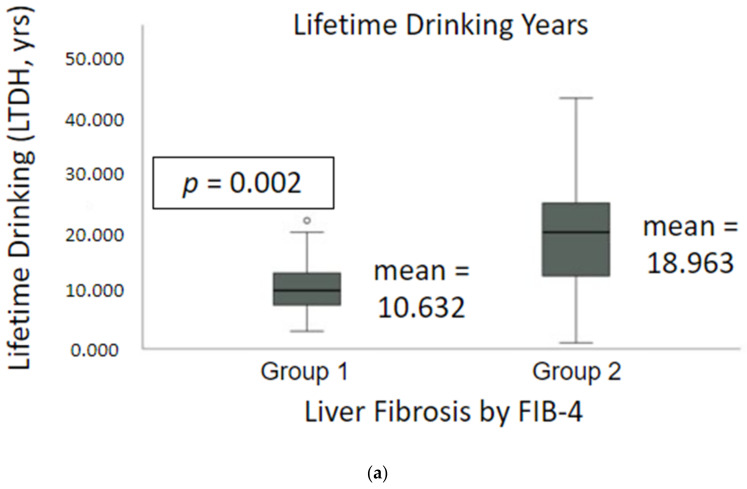

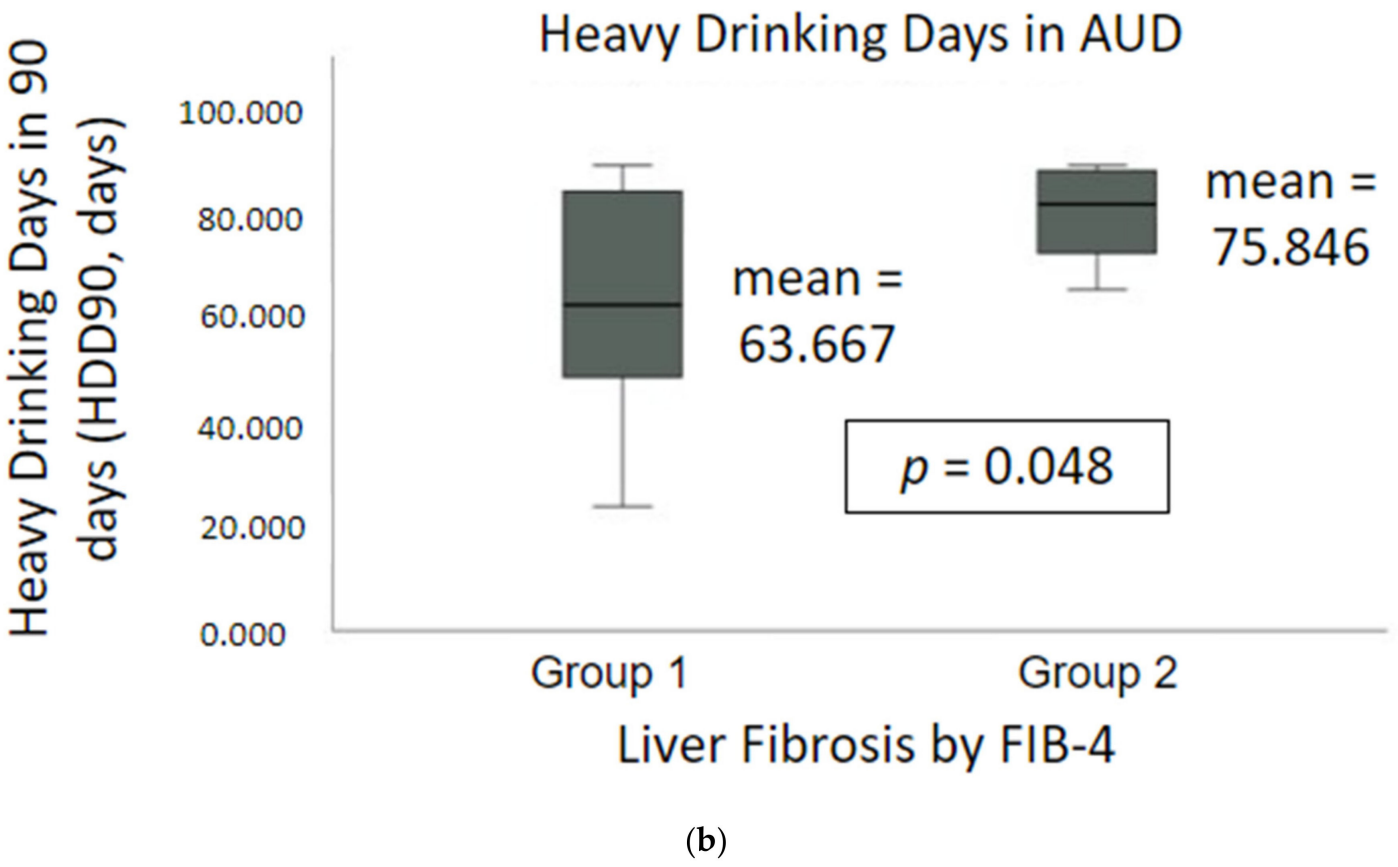
Drinking history in alcohol use disorder patients in Gr.1 (AUD without fibrosis) and Gr.2 (AUD with fibrosis). (**a**) Lifetime drinking years (LTDH) in Gr.1 vs. Gr.2; (**b**) Number of heavy drinking days in the past 90 days in Gr.1 vs. Gr.2. ° outlier values.

Gr.2 (high FIB-4) patients were significantly older compared to Gr.1 patients (low FIB-4); Gr.2 males were significantly older than Gr.2 females (Table 1). BMI was significantly decreased in Gr.2 compared to Gr.1, with unique gender-differences within and between the sub-groups (Table 1). LTDH was significantly elevated in Gr.2 by approximately 2-fold (Figure 2a). HDD90 was also significantly elevated in Gr.2 vs. Gr.1 (Figure 2b). In addition, HDD90 was higher in males versus females of Gr.1, but such a large difference is not seen in Gr.2. TD90, AvgDPD90, and NDD90 were not significantly different between the two groups. CONUT value was also almost 2-fold higher in Gr.2 compared to Gr.1.

Among patients with baseline elevated FIB-4, patients in Gr.2d (FIB-4 > 3.25) demonstrated no significant differences in age or BMI when compared to Gr.2c (1.45 ≤ FIB-4 ≤ 3.25) (Table 2). Gr.2d did have a significantly higher HDD90 and a significantly lower LTDH (Table 2). There were also no significant differences in CONUT.

### 2.2. Liver Injury and Liver Cell Death Markers

**Figure 3:**
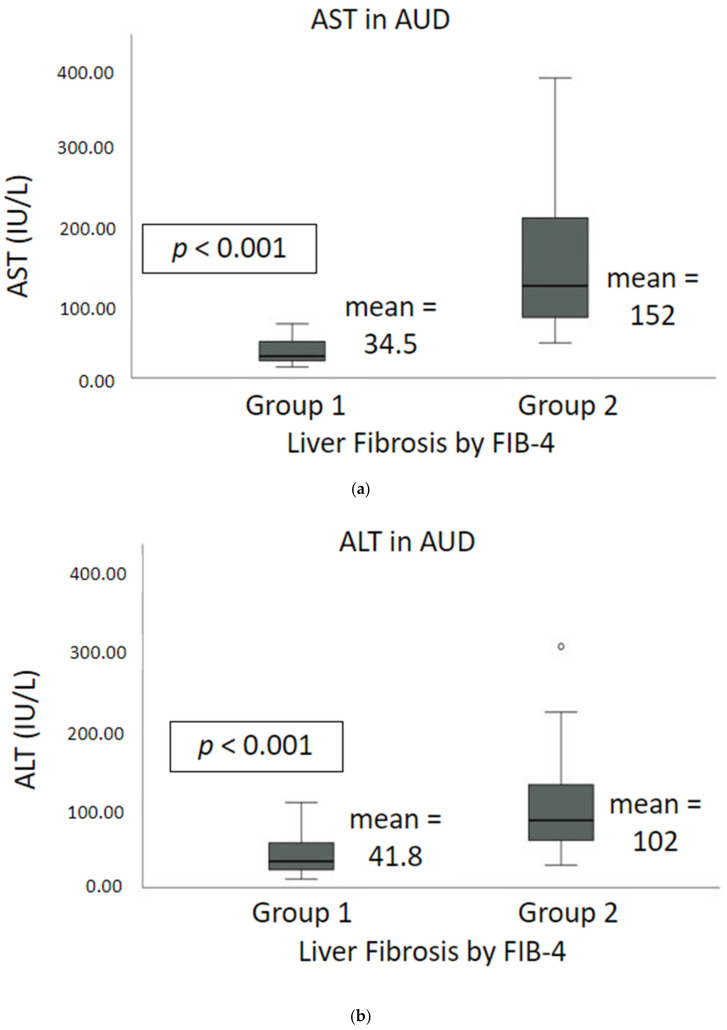

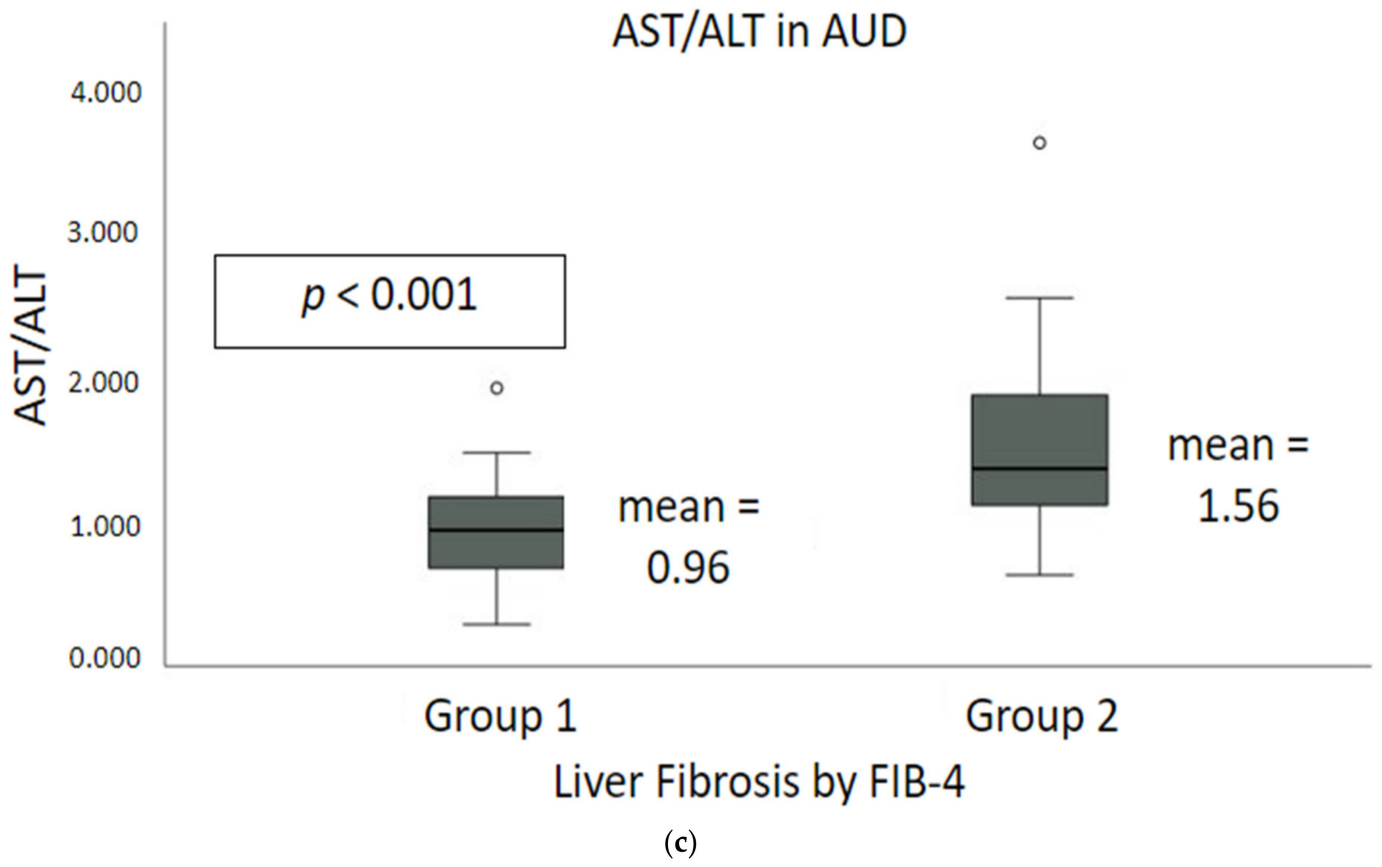
Liver injury markers: AST, and ALT in alcohol use disorder (AUD) patients in Gr.1 compared to Gr.2. (**a**) AST; (**b**) ALT; (**c**) AST/ALT, respectively, in AUD patients without versus with fibrosis. ° outlier values.

ALT, AST, and AST/ALT were significantly elevated in Gr.2 compared to Gr.1 (Figure 3). AST was over 4-fold elevated in Gr.2 compared to Gr.1 (Figure 3a). ALT in Gr.2 was around 2.5-fold elevated compared to Gr.1 (Figure 3b). AST/ALT was also elevated in Gr.2 (Figure 3c).

K18M65 was also significantly higher by approximately 5-fold in Gr.2 compared to Gr.1, *p* < 0.001. K18M65:M30 was significantly elevated in Gr.2 compared to that in Gr.1, *p* < 0.001. Finally, K18M30 was elevated in Gr.2, with a trend towards significance (Table 1).

Among patients with baseline elevated FIB-4, patients in Gr.2d (FIB-4 > 3.25) demonstrated with significance a two-fold higher AST level when compared to patients in Gr.2c (1.45 ≤ FIB-4 ≤ 3.25) (Table 2). There were no clinically or statistically significant differences demonstrated among any of the liver cell death markers (Table 2).

### 2.3. Gut dysfunction and Proinflammatory Response

LPS was significantly higher in Gr.2 compared to Gr.1. LBP was also numerically higher in Gr.2 compared to Gr.1, though this was not statistically significant likely due to the high standard of error (Table 1). TNF-α, one of the earliest active cytokines in the pro-inflammatory cascade in ALD [13], was significantly elevated in Gr.2. IL-6 and IL-8, which are also active pro-inflammatory cytokines in this cascade, were numerically higher in Gr.2 patients (Table 1). IL-6 in Gr.2 was almost two-fold higher compared to IL-6 in Gr.1. While significance was not tested between the genders (due to low numbers of females), it should be noted that numerically IL-8 was higher in females in both the groups (Table 1).

Among patients with a baseline elevated FIB-4, patients in Gr.2d (FIB-4 > 3.25) demonstrated no significant differences in gut dysfunction markers when compared to patients in Gr.2c (1.45 ≤ FIB-4 ≤ 3.25) (Table 2). Gr.2d patients did have significantly higher IL-6 (*p* = 0.003) and IL-8 (*p* = 0.023) levels (Table 2).

### 2.4. Improvement in Immune and Liver Cell Death Status after Treatment in AUD Patients with Fibrosis

**Figure 4:**
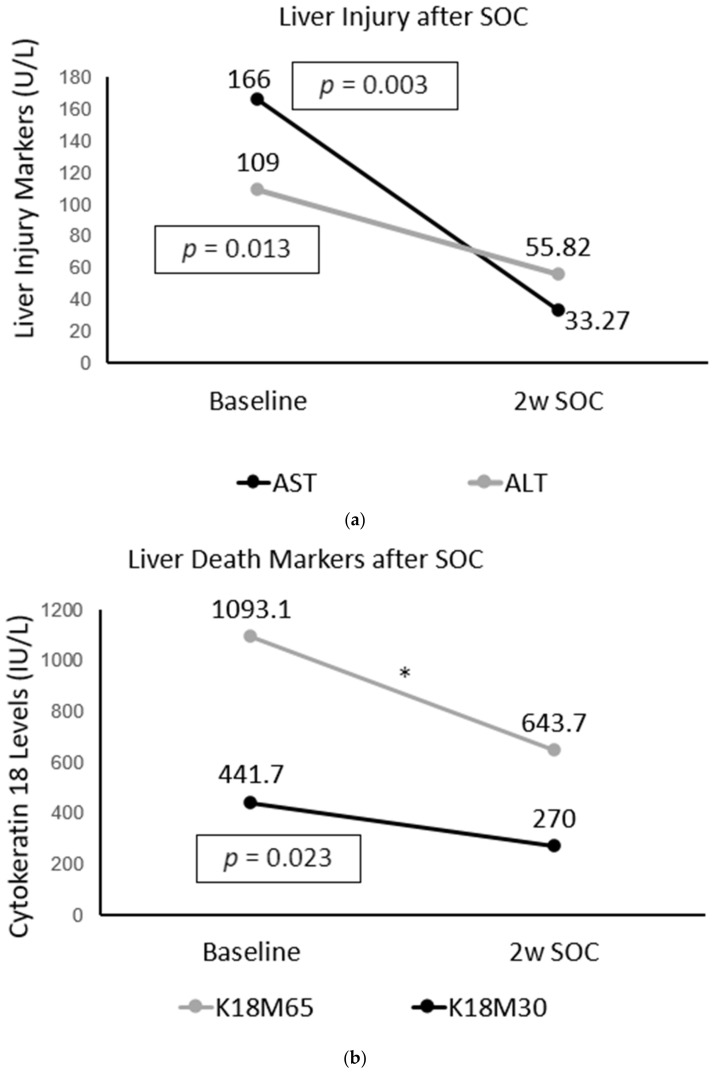
Liver injury and cell death markers after 2 weeks of standard of care (2w SOC). (**a**) AST and ALT after 2w SOC; (**b**) Cytokeratin K18M65 (marker of hepatocyte necrosis) and K18M30 (marker of hepatocyte apoptosis) after 2w SOC. * Not statistically significant.

After 2 w SOC, Gr.2 had significant improvements in liver injury. AST (*p* = 0.003) and ALT (*p* = 0.013) decreased significantly (Figure 4a). Notably, the progression marker AST/ALT ratio also decreased overtime significantly, *p* ≤ 0.001 (Table 3). K18s demonstrated similar improvements after SOC as well. K18M65 decreased after 2 w SOC, although not with statistical significance (Figure 4b). K18M30 (*p* = 0.023) and K18M65:M30 ratio (*p* = 0.001) significantly decreased over time.

Among patients with baseline elevated FIB-4 and after 2 w SOC, patients in Gr.2c (FIB-4 >3.25) had a significantly elevated LBP when compared to patients in Gr.2c (1.45 ≤ FIB-4 ≤ 3.25) (Table 4). The remaining measures were all not statistically significant between the two groups, but there were some unique gender differences within and between the two groups. In addition, K18M65 was decreased by approximately 2-fold in Gr.2d when compared to Gr.2c (Table 4). There were minimal differences in K18M30 and M65:M30.

**Figure 5:**
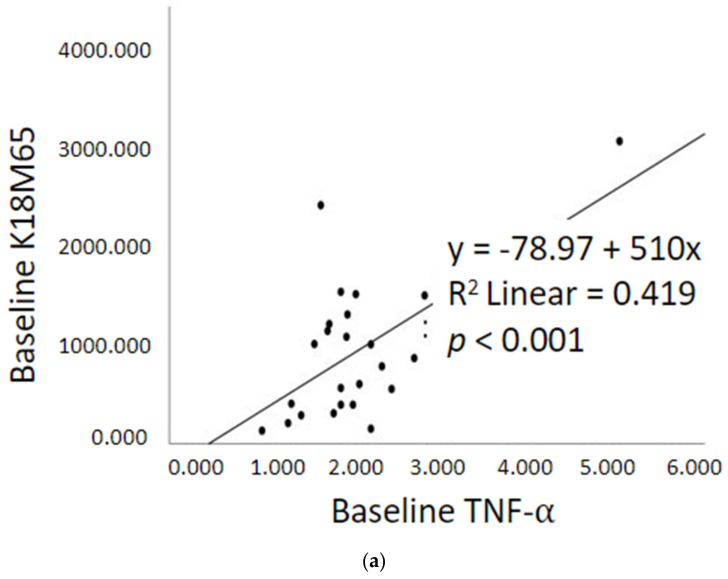

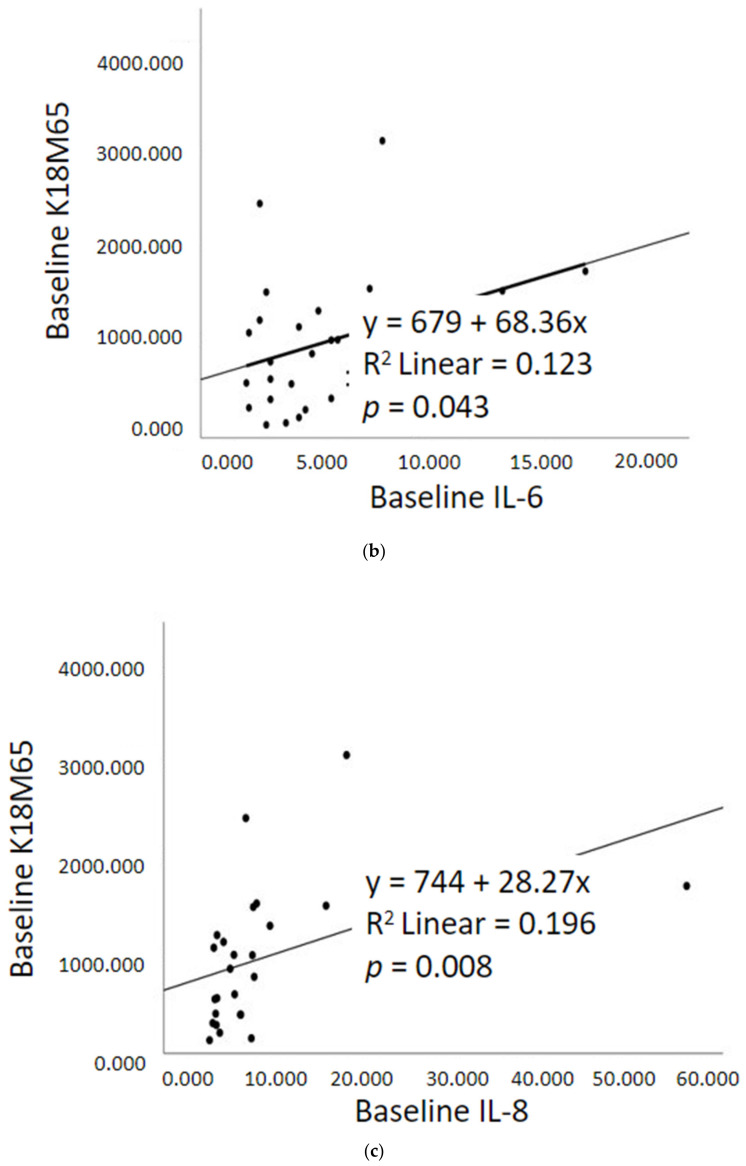
Univariate regression analyses of cytokines with K18M65 within Gr.2—AUD patients with fibrosis (**a**) K18M65 compared with baseline TNF-α; (**b**) with baseline IL-6; and (**c**) with baseline IL-8.

Within Gr.2 patients, multivariate regression analyses demonstrated several significant correlations between pro-inflammatory cytokines and liver cell death markers. In Gr.2 patients, baseline TNF-α and baseline IL-6 were each significantly correlated with K18M65 (r = 0.647; r = 0.350, respectively, at *p* < 0.05) (Figure 5a,b). In addition, the two markers combined also significantly correlated with K18M65 when analyzed with stepwise regression (r = 0.652, *p* < 0.01). BL IL-8 was also significantly associated with BL K18M65 (Figure 5c).

### 2.5. Prognostic Assessment of Immune Response and Liver Cell Death after Treatment

**Figure 6:**
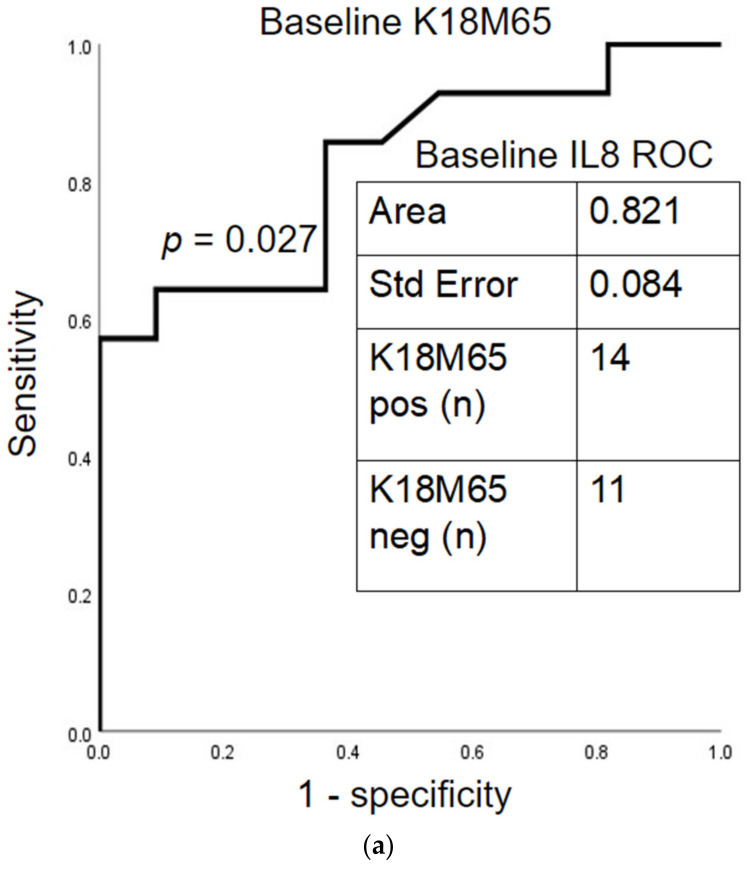

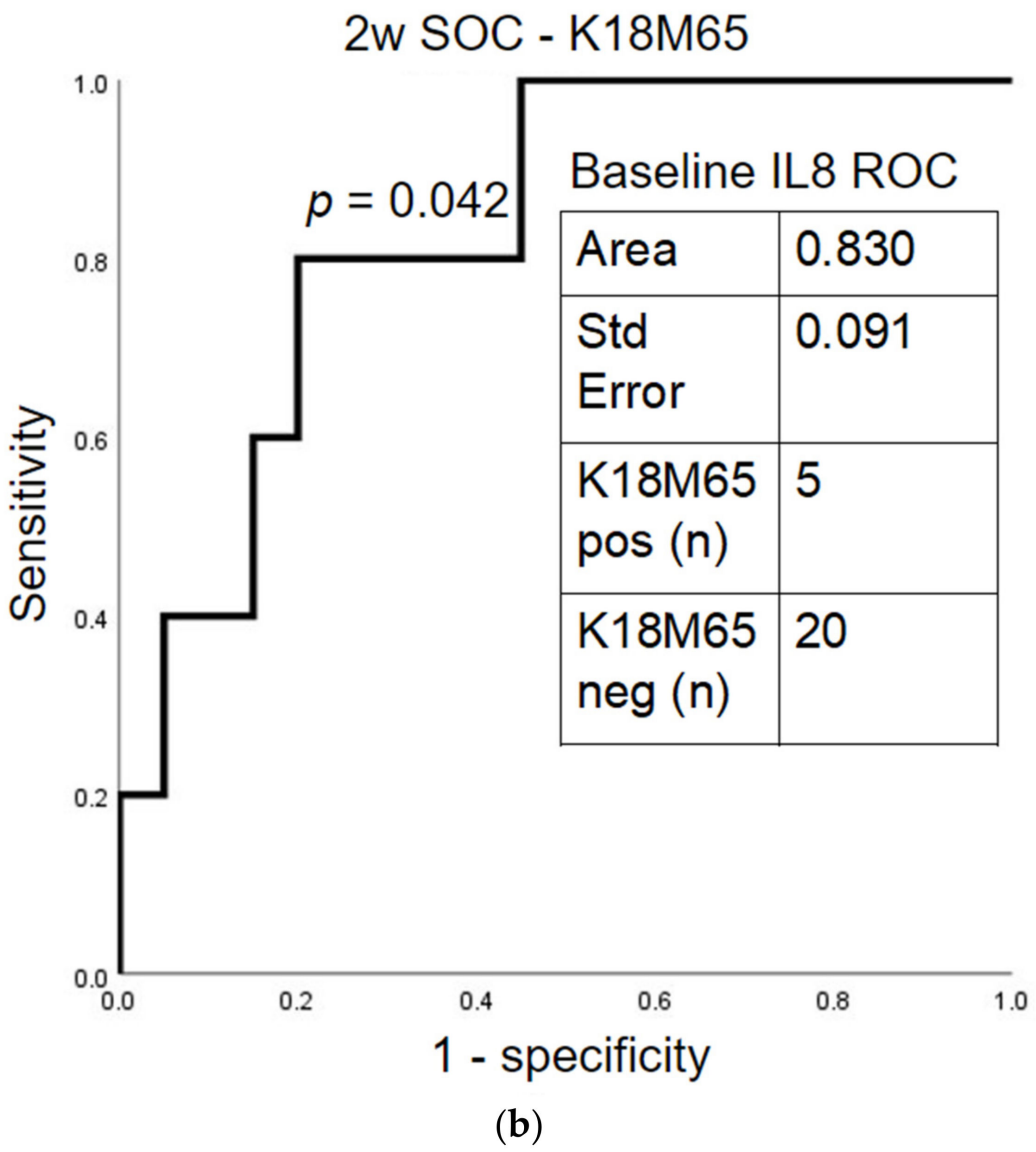
Theragnostic prediction efficacy of 2-week inpatient standard of care in alcohol use disorder patients for improvement in K18M65 in Gr.2. (**a**) True positivity of baseline IL-8 response in predicting K18M65 improvement to normal levels using AUROC Analyses with baseline; (**b**) and after 2 weeks of standard of care. K18M65 as 500 IU/L as normal was used as the primary factor.

**Figure 7:**
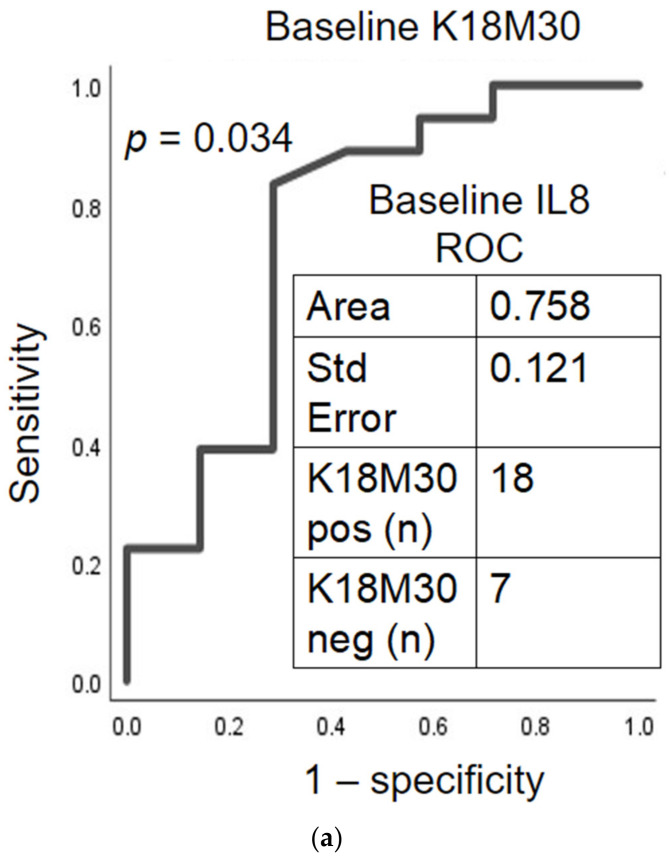

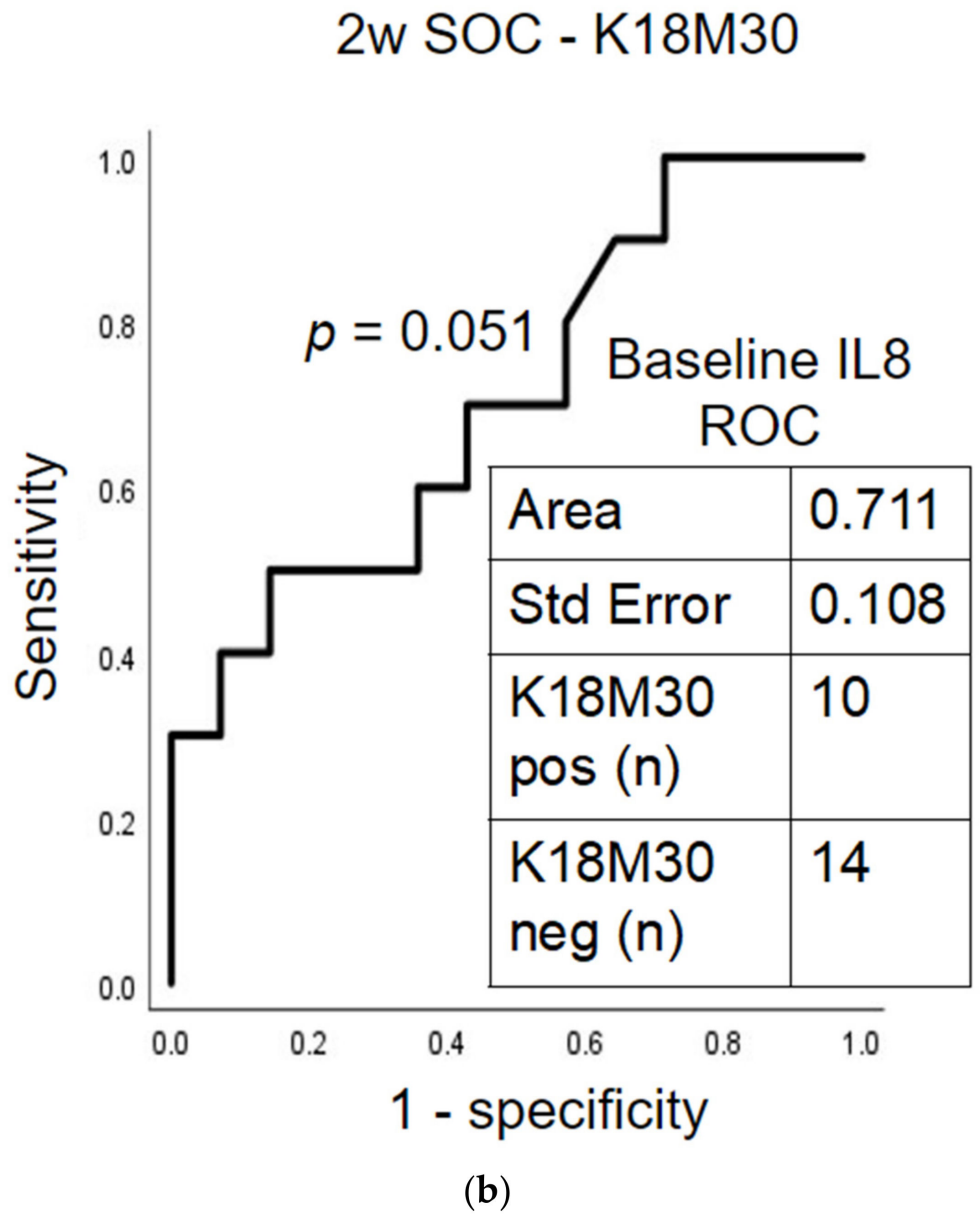
Theragnostic prediction efficacy of 2-week inpatient standard of care in alcohol use disorder patients for improvement in K18M30 in Gr.2. (**a**) True positivity of baseline IL-8 response in predicting K18M30 improvement to normal levels using AUROC Analyses with baseline; (**b**) after 2 weeks of standard of care. K18M30 as 250 IU/L as normal was used as the primary factor.

BL IL-8 particularly had significant predictive efficacy of necrotic liver cell death by the factor of BL K18M65 (AUROC = 0.821 at *p* = 0.027) in Gr.2. Notably, BL IL-8 further predicted the improvement in K18M65 to <500 IU/L at 2 w SOC, *p* = 0.042 in Gr.2 as well (Figure 6a,b). BL IL-8 also could predict the change in apoptotic type of liver cell death (by the incident factor of K18M30, which has also been associated with fibrosis) [14] at baseline and after 2 w SOC with high true positivity (Figure 7a,b). With such findings, we reviewed the baseline values of Gr.2 patients by factoring those with improvement (K18M65 < 500 IU/L) against those who did not improve (K18M65 ≥ 500 IU/L), as Gr.2a and Gr.2b, respectively.

Chi-squared analysis was conducted to compare the distribution of patients by the factors of BL and 2 w K18M65, and BL FIB-4 categories in all the study participants. The majority (72.4%) of patients at baseline with negative K18M65 also had no fibrosis; on the other hand, patients at baseline who had elevated K18M65, also exhibited fibrosis (*n* = 19 out of 21). This interaction was highly significant (*p* < 0.001), with a very high likelihood ratio of 31.628 (largely elevated category). Notably, after 2 w SOC, all of the patients who did not have improvement in K18M65 also had elevated BL FIB-4 (100%, *n* = 7). Half of the patients with K18M65 improvement to less than 500 IU/L had normal BL FIB-4. This interaction was significant (*p* = 0.003), with a high likelihood ratio of 8.658 (borderline highly elevated category).

### 2.6. Baseline Differences in AUD Patients with Fibrosis by Their Post-SOC Improvement in K18M65 Levels

There were distinct BL statistically significant differences between AUD patients with underlying clinical fibrosis, who improved from ongoing liver cell death and liver injury (Gr.2a) and those who did not improve (Gr.2b) post 2 w SOC (Table 5). The progression factor, AST:ALT, was significantly elevated in Gr.2b at BL by almost 2-fold. Correspondingly, Gr.2b patients also showed a higher surge in BL IL-6 and IL-8 (by over 2-fold compared to that reported in Gr.2a).

After 2 w SOC, the differences between the AUD patients who had improved K18M65 levels back to normal levels versus those who did not also show defining characteristics. Even though the liver injury markers showed no outward differences, all the liver cell death markers were higher in Gr.2b (Table 3). We found that LBP decreased significantly in Gr.2a by more than 4-fold compared to Gr.2b. Lastly, both IL-6 (more than 2-fold) and IL-8 (more than 2-fold) again showed lasting significant pro-inflammatory signature in Gr.2b (Table 3) compared to Gr.2a.

## 3. Discussion

The development of ALD has been reported with chronic and heavy alcohol drinking in several studies [15,16]. Public health studies by the World Health Organization report an overall increase in consumption of alcohol worldwide and in the United States. In the USA, this has resulted in a corresponding 65% increase in mortality due to liver cirrhosis [17]. A large study on 7000 participants reports that 30 g or more of alcohol intake is the risk threshold for developing cirrhosis and non-cirrhotic liver damage; this risk also increases proportionally with increasing daily intake of alcohol [18], and with modifiers of ALD [19,20] These modifying factors include but are not limited to: gender, ethnicity, smoking status, and BMI [20]. Males and females have differences in the absorption and metabolism of alcohol; females have a lower proportion of body water and can achieve higher concentrations of blood alcohol [20]. BMI is also a significant and well-known modifying factor, with excess fat and elevated free fatty acids in patients with ALD leading to inflammation and further liver injury [20]. Our study demonstrated similar findings in the patients with early-stage ALD who exhibited fibrosis at admission. They displayed significantly elevated lifetime drinking years as well as frequency of heavy drinking days. In addition, there were numerous significant between-gender differences noted throughout.

Most importantly, our study demonstrated significant differences in key biomarkers between the AUD patients with and without fibrosis. There are already basic markers of liver injury, such as AST, ALT, alkaline phosphatase (ALP) and total bilirubin. However, these are nonspecific and can indicate multiple irregularities including inflammation, poor biliary flow, intrahepatic or extrahepatic disorders [21]. In addition, AST can originate from several sites beyond the liver, such as the heart skeletal muscle, kidney, brain, and red blood cells [21]. ALP on the other hand can also originate from bone, intestine, and placenta in addition to the liver [21]. This increases the need for more targeted markers in analyzing particularly cell death in the liver. There are several studies that analyze K18 levels in liver disease, though primarily in more severe presentation of alcohol-associated hepatitis. Our study is novel as we focused on K18 in early-stage liver disease. We hypothesized that even in early-stage liver disease, heavy and chronic alcohol intake could drive apoptosis and necrosis, leading to elevated levels of circulating cytokeratin fragments in the blood. We found that the baseline K18M65 levels was at a clinically significantly level in most of the AUD patients with fibrosis. After two weeks of standard of care treatment, M65 levels remained at clinically significant levels in Gr.2; albeit M30 levels declined, suggesting lowering of fibrosis [14] as discussed in the supplement section. This supports the hypothesis that the liver still undergoes both apoptosis and necrosis in early damage; however, in more severe disease, for example in which patients did not improve after SOC, there is a decrease in liver function and its ability to undergo a more controlled death, and necrosis becomes more prevalent. Elevated K18 levels have been associated with liver damage in several reports on ALD. Severe alcohol-associated hepatitis and alcohol-associated cirrhosis both have demonstrated markedly elevated K18 fragments [22,23]. In addition, in NASH as well as in non-NASH, both forms of K18 correlate significantly with the degree of steatosis, lobular inflammation, and ballooning [24].

We also found several key pro-inflammatory drivers of inflammation of the liver observed in ALD that were significantly correlated with K18M65 levels. Consumption of alcohol leads to increased permeability of the intestinal membrane. This releases the endotoxin LPS into the portal flow causing activation of Kupffer cells, which yield pro-inflammatory cytokines such as TNF-α, IL-6, IL-8, and IL-1β [25]. TNF and interleukin-8 production is very high in ALD [26]; we found our results consistent. TNF-α in particular activates various signal transduction pathways responsible for cell proliferation, inflammatory responses, and cell death (both apoptotic and necrotic) [27]. In fact, one study demonstrates that excessive alcohol treatment to TNF-α knockout mice does not cause any liver injury, making it likely the primary cytokine responsible for inflammation [28]. TNF-α in turn stimulates production of IL-8, among others, by hepatocytes, a critical chemokine that is responsible for attracting neutrophils and their infiltration into the liver, enhancing inflammation [29]. A high IL-6 response is indicative of induction of Th-17 cell proinflammatory activity [30]. IL-1β is a key cytokine for the commitment of Th-17 cells to synthesize IL-17 proinflammatory cytokine [31]. Such cytokines further enhance inflammation by promoting differentiation of Th-17 cells and therefore production of IL-17. This in turn stimulates Kupffer cells as well as hepatic stellate cells to produce pro-inflammatory cytokines, and chemokines. These activated stellate cells differentiate into myofibroblasts, which promote fibrosis in the liver [32]. TNF-α can also bind to type 1 TNF receptor1 (TNFR-1) to recruit intracellular proteins that are involved in inducing apoptotic cell death, partially through activation of caspase-8 [7,33,34]. In our study, gut dysfunction and elevated immune activity were highly evident in the AUD patients with positive fibrosis status than those without fibrosis, specifically by the elevated levels of TNF-α, IL-8, and LPS. These pro-inflammatory drivers also showed greater affinity with the elevated markers of necrosis in the liver, particularly TNF-α and IL-6.

After two weeks of SOC, we found that there was a significant improvement in liver injury in Gr.2, with significantly lower AST, ALT, and AST/ALT. This coincided significantly with a decrease in necrosis as well as apoptosis markers, with large simultaneous reductions in K18M65, K18M30, and K18M65:M30 ratio values. AUC analysis was also utilized to determine if any of the significantly associated cytokines had an ability to estimate the change in K18 response after 2 w SOC. Baseline IL-8 specifically presented a significant ability to predict K18s improvement after SOC. Such robust predictive efficacy signifies that even at baseline, a pro-inflammatory cytokine individually can predict not only elevated liver cell death in the fibrosis positive group but can also observe the improvement in disease after SOC. Similar findings are found in studies on chronic liver failure in patients with chronic hepatitis B virus. One study reports that both M65 and M30 levels increase as liver disease progresses, while the prognostic value of M30/M65 ratio in predicting the improvement in liver disease is high with a specificity of 92.6% [35].

Most of the study patients with baseline fibrosis improved after 2 weeks of SOC while others uniquely did not. We found patients who did not have improvement in K18M65 levels had higher levels of all the drinking indices at baseline compared to those who improved. In addition, there was greater inflammation particularly later in the inflammatory cascade in the group without improvement. IL-6 and IL-8, which are considered the outcome of the stimulation by TNF-α, were notably higher. As these labs were checked after two weeks of SOC, this is consistent with the delayed cytokine response.

Primarily, every patient that had a positive baseline K18M65 also had an elevated FIB-4 score at baseline. In addition, after 2 weeks SOC, every patient that did not have improvement in K18M65 also had an elevated FIB-4 at baseline, all of which strongly suggests the presence of necrosis should be taken very importantly in the early liver disease with fibrosis. Patients with similar drinking and K18 levels could very likely progress to an advanced form of ALD [36]. K18M30 levels in 10 out of 18 patients with baseline fibrosis could not lower to the normal levels; this sub-cohort ultimately might continue to progress with fibrosis and could show exacerbation as advanced ALD. Thus, patients with AUD exhibiting fibrosis in the liver is a risky cohort that could decompensate overtime compared to those who did not exhibit fibrosis, regardless of the levels of the liver injury markers.

Just like any other study, our study also had a number of limitations. The FIB-4 score is inferior to the gold standard of liver biopsies and elastography; albeit the direction of this study was to identify a convenient and non-invasive marker, without the need of advanced technical support. In addition, patients were grouped as either having fibrosis or having no fibrosis based on FIB-4 values of greater than or equal to 1.45 or less than 1.45, respectively. Previous work has shown that FIB-4 < 1.45 has a negative predictive value of 94.5% to exclude severe fibrosis, while FIB-4 ≥ 3.25 had a specificity of 98.2% to predict advanced fibrosis [37]. We also conducted a subgroup analysis within the elevated FIB-4 group to analyze the differences between those with a FIB-4 between 1.45 and 3.25, and those with ≥ 3.25 (Table 5). This analysis found that while there are significant differences in AST and cytokines IL-6 and IL-8 between the two groups, there were no significant differences among any of the cytokeratins of interest. Another limitation of our study is the small sample size for some group comparisons. While our study showed that K18 has promise as a predictive marker for early liver disease progression, larger, prospective studies will need to be conducted to determine its role in predicting the development of liver disease in at risk populations with AUD. Another limitation is that cytokeratins are not part of a traditional lab panel and is likely a send-out lab in most hospitals. Hopefully, as further research demonstrates the usability of these as biomarkers, the testing of these markers becomes widely available. In addition, our study demonstrated the benefit of using a more common cytokine, baseline IL-8, as a significant predictor for the course of liver disease after a SOC treatment. Finally, a CMP was not available for all patients after 2 weeks of SOC, as oftentimes it was not clinically indicated, thus FIB-4 levels were not available for analyses at the 2-week timepoint in this study. However, our focus was on the liver cell death shift over a clinically relevant period in patients with and without baseline fibrosis; K18M30 response was very encouraging along with being strongly associated with fibrosis.

Our findings confirm that even early alcohol-associated liver injury is associated with a significant elevation in inflammatory cytokines as well as the novel biomarker K18 in the blood. This is significant for clinicians as only a subset of patients with alcohol use disorder progress to liver injury and disease, and this subset is difficult to predict. Our work adds to the growing body of literature that K18 is a promising biomarker for liver injury, with potential to identify at-risk patients with AUD which have higher likelihood to progress towards the advanced form of ALD.

## 4. Materials and Methods

### 4.1. Human Subjects

This clinical study was one of the secondary aims of a large umbrella clinical protocol (ClinicalTrials.gov identifier # NCT00106106) that was conducted in the National Institute on Alcohol Abuse and Alcoholism (NIAAA) at the National Institutes of Health (NIH), Bethesda MD. The study protocol was approved by the Institutional Review Board of the central neuroscience committee of the NIAAA. Study subjects selected were those who were seeking study-based treatment as described in the clinical protocol, which included a 28-day admission to the NIAAA in-patient facility (Clinical Center, NIH) for alcohol detoxification with standard of care treatment. All eligible patients seeking treatment consented to participate in the study before study-based clinical and research data, and research related bodily samples were collected. Screening was performed on the potential participants to test if they met the requirements of the study eligibility and exclusion criteria.

Main eligibility criteria were as follows: all study patients were diagnosed with AUD based on DSM-IV TR edition [38]. The alcohol dependence module of the structured clinical interview and alcohol withdrawal were used for determining the diagnosis AUD in the potential participants. Eventually, 48 male and female AUD patients between 21 and 65 years of age participated in this study from a larger protocol. Exclusion criteria for this study were: diagnosis of severe psychiatric and/or somatic illnesses, such as advanced lung disease, unstable cardiovascular disease (decompensation, as demonstrated through chest X-ray, pathological electrocardiogram), and/or renal failure (creatinine clearance <30 mL/min). Other study-specific exclusion criteria included: HIV-positive, pregnancy or ongoing breastfeeding, pronounced anxiety provoked by enclosed spaces, and/or positive urine screen for any illicit drug. No AUD patient exhibited any clinical evidence of advanced ALD. Several of our studies further detail the information on admission, exclusion and inclusion, and detox treatment [15,39,40,41]. Patients were screened during the first three days of admission, in which they were decided to be part of the SOC group or to be eligible for randomization in studies. Study patients during the course of inpatient stay received standard of care (SOC) for alcohol detoxification (specific domains of AUD; primarily withdrawal and craving) [42] and medical management (metabolic, nutritional), including counseling according to the “Human Subjects Protection” guideline of NIH. SOC included obtaining a medical history and physical examination on each patient, neurological evaluation, laboratory tests, nutrition, discharge planning and referrals for treatment of conditions if needed. Study patients may have received acamprosate versus a placebo as part of the overarching NIH study. Patients also may have received diazepam for treatment of severe withdrawal symptoms (ClinicalTrials.gov identifier # NCT00106106). Both acamprosate and diazepam do not have any interaction with liver function or influence liver health, thus these patients were included within this study [43,44]

The FIB-4 score was used to estimate the severity of scarring in the liver [45]. One study demonstrates that a FIB-4 score <1.45 has a negative predictive value of 90% for advanced fibrosis in hepatitis C [45]. Another study demonstrates a high diagnostic power of FIB-4 for fibrosis in patients with AUD, with an AUC of 0.948 for a FIB-4 > 1.24 [46]. A FIB-4 score > 3.25 is also demonstrated to have a specificity of 97%, particularly in patients with HIV/HCV coinfection [45]. Our study design stratified the patients with AUD into two groups—Group 1 (Gr.1) with clinically insignificant (<1.45) FIB-4 and Group 2 (Gr.2) with clinically significant (≥1.45) FIB-4. We also conducted a subgroup analysis within Gr.2 with the higher cut-off of 3.25 to evaluate for any differences in the two cut-off values.

### 4.2. Demographic, Alcohol Intake and Laboratory Evaluations

Measurements of demographics (age, gender, BMI), chronic and recent alcohol intake were collected (Table 1). Alcohol intake for the past 90 days (Timeline Follow-back Instrument) was used for recent drinking history [47]; and the Lifetime Drinking History (in yrs.) assessment was utilized for chronic misuse [48]. Drinking markers derived from Timeline Followback were: Total Drinks past 90 Days (TD90), Heavy Drinking Days Past 90 Days (HDD90), Number of Drinking Days Past 90 Days (NDD90), Average Drinks Past 90 days (AvgD90) [39,42]. More information on the collection of this drinking history information has been reported comprehensively in our several previous publications [42,49,50]. CONtrolling NUTritional status [CONUT or CAL score] was also reported. This was derived from the categorical levels of serum total serum cholesterol (mg/dL), serum albumin (g/dL), and total lymphocyte count (/mL of blood). This was performed to estimate nutritional deficiency [51]. All laboratory assays were analyzed with the guideline set-up at the Medline Plus (set until 2014, when the clinical samples were assayed) at the Department of Laboratory Medicine of the National Institutes of Health.

### 4.3. Laboratory Analyses

Multianalyte chemiluminescent detection was performed for cytokine assays (TNF-α, IL-1β, IL-6, and IL-8, plasminogen activator inhibitor 1 [PAI-1], and monocyte chemoattractant protein-1 [MCP-1]) using Multiplex kits (Millipore, Billerica, MA, USA) on the Luminex (Luminex, Austin, TX, USA) platform based on the manufacturer’s guidelines. K18 whole protein (K18M65), which reflects hepatocyte necrosis, and caspase-cleaved fragment (K18M30), which reflects hepatocyte apoptosis, were tested by the enzyme-linked immunosorbent assay (ELISA) (Peviva-VLVbio, Nacka, Sweden) following the manufacturer’s recommendations. The clinical range level of K18 is as follows: K18M65 ≥ 500 U/L; or K18M30 > 250 U/L. LPS, LBP and sCD14 levels were tested using Kinetic Chromogenic limulus Aoebocyte lysate Assaty (Lonza, Walkersville, MD, USA), per manufacturer recommendations.

### 4.4. Statistics

Univariate analysis of covariance (ANCOVA) was used to evaluate differences in the demographic characteristics, drinking history measures, and liver injury markers between the two groups factored by FIB-4. ANCOVA was also used to evaluate differences in liver injury markers, both overall and by gender as a factor within each group as well as between the two groups. We also conducted a separate analysis within Gr.2 (elevated FIB-4) between patients with 1.45 ≤ FIB-4 ≤ 3.25 and those with FIB-4 > 3.25 to evaluate for any significant differences between the cut-off values. This was done at both baseline (Table 2) and after 2w SOC (Table 4). The gut-derived proinflammatory response was correlated with liver injury and proinflammatory markers using a univariate and multivariate regression paradigm. Drinking history measures were added to the aforementioned univariate and multivariate analyses as secondary independent variables further stratified by gender. Receiver operating characteristic (ROC) curves were constructed to determine the sensitivity and specificity of the candidate markers (baseline values at admission) to predict the improvement in K18M65 after 2 w of SOC. SPSS 27.0 (IBM, Chicago, IL, USA) and Microsoft office 365 Excel (MS Corp, Redmond, WA, USA) were used for statistical analysis and data computation. Statistical significance was established at *p* ≤ 0.05. Data are expressed as the Mean ± SD (standard deviation) in the tables. An independent samples t-test was then utilized to compare a sub-group of FIB-4 positive patients demonstrating an improved K18M65 (Gr.2a) after 2 w SOC compared to those who did not improve (Gr.2b) (Table 3). Demographics, drinking history, injury markers, cytokines, etc were all compared between the two sub-groups in Gr.2 improvement analyses as well to identify the unique differences. We also tested the key differences between sub-group 2a and 2b for their post 2 w SOC (Table 4). This analysis was also done to compare a sub-group of FIB-4 positive patients with moderate elevation in FIB-4 (1.45 ≤ FIB-4 ≤ 3.25) (Gr.2c) with those with high elevation in FIB-4 > 3.25 (Gr.2d). This was done at both baseline (Table 2) and after 2w SOC (Table 4).

## 5. Conclusions

The findings in this study support the role of a novel biomarker, K18M65, as a predictor for early liver damage and fibrosis in AUD patients. We found that AUD patients who exhibit fibrosis have markers of gut dysfunction, dysregulation of cytokines, and liver cell death similar to those with advanced severe disease. Cytokeratin 18 shows promise as a diagnostic predictor in AUD patients who have early-stage ALD with fibrosis.

## Figures and Tables

**Table 1:** Baseline demographics, drinking history, liver injury measures, nutritional status, candidate blood panel measures, cytokines, gut dysfunction markers, and cell death markers of the alcohol use disorder patients tabulated by FIB-4 present/absent criteria.

Measures	Group 1 (Normal FIB-4, Gr.1)			Group 2 (Elevated FIB-4, Gr. 2)			Between Group *p*-Value
	Males (*n* = 15; 71.4%)	Females (*n* = 6; 28.6%)	Total (*n* = 21; 43.8%)	Males (*n* = 19; 70.4%)	Females (*n* = 8; 29.6%)	Total (*n* = 27; 56.3%)	
Age ^d^ (years)	37.6 ± 10.3	39.6 ± 11.1	38.2 ± 10.3	47.8 ± 7.0	45.5 ± 12.6	47.1 ± 8.8	0.002
BMI ^abc^ (kg/m^2^)	28.5 ± 5.1	30.1 ± 11.1	29.0 ± 7.1	24.9 ± 3.0	25.6 ± 3.8	25.1 ± 3.2	0.024
Drinking History							
TD90	1249 ± 5	855 ± 662	1136 ± 648	1094 ± 482	1027 ± 611	1076 ± 508	ns
HDD90	68.9 ± 20.7	50.7 ± 17.2	63.7 ± 21.1	74.7 ± 21.2	78.9 ± 16.9	75.8 ± 19.9	0.048
AvgDPD90	16.5 ± 6.0	15.7 ± 8.5	16.2 ± 6.6	14.7 ± 5.6	12.3 ± 6.4	14.0 ± 5.8	ns
NDD90	73.2 ± 17.2	52.0 ± 17.2	67.1 ± 19.4	76.5 ± 20.4	80.6 ± 14.1	77.6 ± 18.7	ns
LTDH ^b^	12.1 ± 5.6	7.5 ± 2.6	10.6 ± 5.2	21.6 ± 10.1	12.8 ± 8.2	19.0 ± 10.3	0.002
Liver Injury Markers							
ALT ^abcd^ (IU/L)	51.3 ± 24.0	18.2 ± 5.3	41.8 ± 25.4	99.3 ± 37.9	110 ± 102	102 ± 62	*p* < 0.001
AST ^abd^ (IU/L)	38.2 ± 18.4	25.2 ± 12.2	34.5 ± 17.6	140 ± 82	180 ± 133	152 ± 99	*p* < 0.001
AST: ALT ^a^	0.80 ± 0.28	1.36 ± 0.35	0.96 ± 0.39	1.43 ± 0.57	1.87 ± 0.87	1.56 ± 0.68	*p* < 0.001
Nutritional Status							
CONUT^ad^	0.60 ± 0.63	0.83 ± 1.17	0.67 ± 0.80	1.42 ± 1.47	0.75 ± 0.71	1.22 ± 1.31	ns
Blood Cell Measures							
WBC ^a^ (K/uL)	7.57 ± 2.91	7.99 ± 2.77	7.69 ± 2.81	4.95 ± 1.57	7.01 ± 1.89	5.56 ± 1.89	0.004
AMC (K/uL)	0.65 ± 0.28	0.51 ± 0.13	0.61 ± 0.25	0.45 ± 0.23	0.49 ± 0.08	0.46 ± 0.19	0.026
ANC ^a^ (K/uL)	4.38 ±2.40	4.71 ± 1.90	4.48 ± 2.22	2.95 ± 1.23	4.36 ± 1.92	3.37 ± 1.57	0.049
Candidate Cytokine Response							
IL1β (pg/mL)	0.52 ± 0.29	0.52 ± 0.46	0.52 ± 0.33	0.52 ± 0.30	0.50 ± 0.60	0.51 ± 0.40	0.002
IL6 (pg/mL)	3.03 ± 2.17	1.40 ± 0.72	2.60 ± 2.01	3.18 ± 2.82	6.61 ± 4.46	4.28 ± 3.71	ns
TNFα ^ad^ (pg/mL)	1.81 ± 0.87	1.35 ± 0.88	1.69 ± 0.87	1.90 ± 0.51	2.39 ± 1.46	2.06 ± 0.92	0.025
IL8 ^acd^ (pg/mL)	2.92 ± 1.27	16.68 ± 31.26	6.54 ± 16.03	5.55 ± 3.52	13.40 ± 19.08	8.06 ± 11.33	ns
Candidate Gut-dysfunction Markers							
LPS ^a^ (EU/mL)	0.082 ± 0.045	0.076 ± 0.054	0.080 ± 0.047	0.114 ± 0.065	0.112 ± 0.062	0.114 ± 0.063	0.050
LBP ^c^ (ng/mL)	926.5 ± 1347	2439 ± 4030	1304 ± 2282	2263 ± 2792	2355 ± 3911	2289 ± 3057	ns
CD14 (×10^6^ pg/mL)	8604 ± 1590	9748 ± 1757	8931 ± 1680	9198 ± 2010	10526 ± 1141	9592 ± 1879	ns
Liver Cell Death Markers							
K18M65 ^ab^ (IU/L)	238.6 ± 119.2	151.6 ± 66.5	213.7 ± 112.6	1115 ± 1018	1040 ± 956	1093 ± 982	*p* < 0.001
K18M30 (IU/L)	202.2 ± 70.7	568.5 ± 923.0	306.8 ± 495.2	407.7 ± 377.8	515.2 ± 335.7	439.5 ± 362.8	ns
M65:M30 ^a^	1.28 ± 0.66	0.68 ± 0.52	1.11 ± 0.67	2.73 ± 1.55	1.90 ± 0.79	2.48 ± 1.41	*p* < 0.001

Significant between group analyses for: ^a^ males only between Gr.1 and Gr.2, ^b^ females only between Gr.1 and Gr.2, ^c^ between gender in Gr.1 only, and ^d^ between gender in Gr.2 only. BMI: Body mass index; TD90: Total drinks past 90 days; HDD90: heavy drinking days past 90 days; AvgDPD90: Average drinks per drinking day past 90 days; NDD90: number of drinking days past 90 days; NNDD90: number of non-drinking days past 90 days, LTDH: lifetime drinking history (in years), ALT: serum alanine aminotransferase, AST: serum aspartate aminotransferase, AST:ALT: ratio of AST by ALT, CONUT: Controlling Nutritional Status Test (unit: numerical), WBC: white blood cell count, AMC: absolute monocyte count, ANC: absolute neutrophil count, IL-1β: interleukin 1 beta, IL-6: interleukin 6, TNF-α: tumor necrosis factor alpha, LPS: lipopolysaccharide, LBP: LPS-binding protein, sCD14: soluble cell of differentiation type 14, K18M65: soluble K18, K18M30: caspase-cleaved fragment of K18, M65:M30: ratio of K18M65 by K18M30.

**Table 2:** Within elevated baseline FIB-4, tabulation by moderate and severe elevation of FIB-4 of: demographics, drinking history, baseline liver injury measures, baseline nutritional status, baseline candidate blood panel measures, baseline cytokines, baseline gut dysfunction markers, and baseline cell death markers of alcohol use disorder patients. Only markers that were significantly different in Table 1 are presented here.

Measures	Group 2c (1.45 ≤ FIB-4 ≤ 3.25), Gr. 2c)			Group 2d (FIB-4 > 3.25, Gr. 2d)			Between Group *p*-Value
	Males (*n* = 10; 71.4%)	Females (*n* = 4; 28.6%)	Total (*n* = 14; 51.9%)	Males (*n* = 9; 69.2%)	Females (*n* = 4; 30.7%)	Total (*n* = 13; 48.1%)	
Age ^c^ (years)	44.84 ± 5.27	41.87 ± 15.06	43.99 ± 8.57	51.05 ± 7.42	49.07 ± 10.46	50.44 ± 8.06	ns
BMI (kg/m^2^)	24.99 ± 3.11	24.30 ± 4.72	24.79 ± 3.45	24.81 ± 2.95	26.83 ± 2.66	25.48 ± 2.91	ns
Drinking History							
TD90	1199 ± 601	1182 ± 821	1195 ± 619	978 ± 296	911 ± 507	958 ± 351	ns
HDD90 ^b^	70.50 ± 25.04	74.00 ± 25.98	71.31 ± 24.19	79.44 ± 16.14	82.50 ± 8.81	80.38 ± 13.97	0.045
AvgDPD90	16.73 ± 6.35	14.36 ± 7.81	16.18 ± 6.44	12.42 ± 3.84	10.77 ± 5.82	11.91 ± 4.35	ns
NDD90	72.10 ± 23.10	77.00 ± 20.78	73.23 ± 21.84	81.33 + 16.83	83.25 ±9.43	81.92 ± 14.56	ns
LTDH ^a^	22.40 ± 6.24	14.75 ± 7.41	20.21 ± 7.24	20.67 ± 13.63	10.75 ± 9.47	17.62 ± 13.00	0.011
Liver Injury Markers							
ALT ^bd^ (IU/L)	97.80 ± 34.93	50.75 ± 23.47	84.36 ± 38.19	100.89 ± 43.12	170.50 ± 119.72	122.31 ± 77.08	ns
AST ^a^ (IU/L)	104.8 ± 49.5	102.3 ± 73.5	104.1 ± 54.3	179.9 ± 94.9	257.8 ± 141.5	203.9 ± 111.4	0.003
AST:ALT	1.064 ± 0.293	1.898 ± 0.476	1.302 ± 0.514	1.830 ± 0.532	1.833 ± 1.235	1.831 ± 0.755	ns
Nutritional Status							
CONUT	0.800 ± 1.135	0.750 ± 0.500	0.786 ± 0.975	2.111 ± 1.537	0.750 ± 0.957	1.692 ± 1.494	ns
Blood Cell Measures							
WBC (K/uL)	5.433 ± 1.333	8.100 ± 1.692	6.195 ± 1.858	4.419 ± 1.710	5.920 ± 1.504	4.881 ± 1.742	ns
AMC ^a^ (K/uL)	0.463 ± 0.130	0.528 ± 0.095	0.481 ±0.121	0.429 ± 0.311	0.452 ± 0.046	3.687 ± 1.762	ns
ANC (K/uL)	3.138 ± 1.216	5.057 ± 2.346	3.687 ± 1.762	2.741 ± 1.293	3.662 ± 1.328	3.025 ± 1.323	ns
Candidate Cytokine Response							
IL1β (pg/mL)	0.618 ± 0.329	0.736 ± 0.794	0.655 ± 0.482	0.409 ± 0.222	0.273 ± 0.242	0.364 ± 0.228	ns
IL6 ^a^ (pg/mL)	2.398 ± 1.022	3.913 ± 1.389	2.864 ± 1.307	4.052 ± 3.916	9.311 ± 5.000	5.804 ± 4.825	0.003
TNFα ^bd^ (pg/mL)	2.049 ± 0.536	1.479 ± 0.0.336	1.874 ± 0.543	1.742 ± 0.456	3.304 ± 1.617	2.263 ± 1.199	ns
IL8 ^d^ (pg/mL)	4.878 ± 1.998	4.154 ± 1.482	4.655 ± 1.825	6.306 ± 4.738	22.637 ± 24.883	11.750 ± 15.742	0.023
Candidate Gut-dysfunction Markers							
LPS ^a^ (EU/mL)	0.124 ± 0.063	0.124 ± 0.081	0.124 ± 0.065	0.104 ± 0.071	0.099 ± 0.046	0.102 ± 0.062	ns
LBP (ng/mL)	2385 ± 3395	723 ± 555	1969 ± 3000	2142 ± 2238	3579 ± 5071	2584 ± 3201	ns
CD14 (×10^6^ pg/mL)	9229 ± 1912	10679 ± 1215	9643 ± 1826	9164 ± 2231	10373 ± 1224	9536 ± 2007	ns
Liver Cell Death Markers							
K18M65 (IU/L)	1124 ± 1281	528 ± 329	953.4 ± 1113.2	1107 ± 696	1552 ± 1151	1244 ± 837	ns
K18M30 (IU/L)	474.7 ± 503.4	404.4 ± 218.3	454.6 ± 433.0	333.1 ± 155.5	626.1 ± 427.2	423.4 ± 285.5	ns
M65:M30	2.232 ± 1.196	1.443 ±0.645	2.007 ± 1.106	3.285 ± 1.780	2.348 ± 0.711	2.997 ± 1.563	ns

Significant between group analyses for: ^a^ males only between Gr.1 and Gr.2, ^b^ females only between Gr.1 and Gr.2, ^c^ between gender in Gr.1 only, and ^d^ between gender in Gr.2 only. BMI: Body mass index; TD90: Total drinks past 90 days; HDD90: heavy drinking days past 90 days; AvgDPD90: Average drinks per drinking day past 90 days; NDD90: number of drinking days past 90 days; NNDD90: number of non-drinking days past 90 days, LTDH: lifetime drinking history (in years), ALT: serum alanine aminotransferase, AST: serum aspartate aminotransferase, AST:ALT: ratio of AST by ALT, WBC: white blood cell count, AMC: absolute monocyte count, ANC: absolute neutrophil count, IL-1β: interleukin 1 beta, IL-6: interleukin 6, TNF-α: tumor necrosis factor alpha, LPS: lipopolysaccharide, LBP: LPS-binding protein, sCD14: soluble cell of differentiation type 14, K18M65: soluble K18, K18M30: caspase-cleaved fragment of K18, M65:M30: ratio of K18M65 by K18M30.

**Table 3:** Liver injury measures, nutritional status, candidate blood panel measures, cytokines, gut dysfunction markers, and cell death markers of the alcohol use disorder patients after 2 weeks of SOC comparing patients with improved K18M65 < 500 IU/L (Gr.2a) and patients with elevated K18M65 ≥ 500 IU/L (Gr.2b) within elevated FIB-4 patients (Gr.2).

Measures	Group 2a (Improved K18M65, Gr. 2a)			Group 2b (Elevated K18M65, Gr. 2b)			Between Group *p*-Value
	Males (*n* = 15; 75%)	Females (*n* = 5; 25%)	Total (*n* = 20; 74.1%)	Males (*n* = 4; 57.1%)	Females (*n* = 3; 42.9%)	Total (*n* = 7; 25.9%)	
Liver Injury Markers							
ALT(IU/L)	61.83 ± 25.21	na	61.83 ± 25.21	51.00 ± 19.00	45.00 ± 25.46	48.60 ± 18.80	ns
AST (IU/L)	29.83 ± 6.31	na	29.83 ± 6.31	39.33 ± 5.03	34.50 ± 6.36	37.40 ± 5.46	ns
AST: ALT	0.55 ± 0.27	na	0.55 ± 0.27	0.85 ± 0.35	0.87 ≠ 0.35	0.86 ± 0.30	ns
Candidate Cytokine Response							
IL1β ^c^ (pg/mL)	0.48 ± 0.23	1.90 ± 1.57	0.87 ± 1.02	0.47 ± 0.23	0.28 ± 0.17	0.39 ± 0.22	ns
IL6 ^ab^ (pg/mL)	2.58 ± 1.02	3.00 ± 0.63	2.70 ± 0.93	6.03 ± 3.99	5.46 ± 1.97	5.78 ± 3.06	*p* < 0.001
TNFα (pg/mL)	2.32 ± 0.70	2.17 ± 0.95	2.27 ± 0.75	2.21 ± 1.10	3.04 ± 1.57	2.56 ± 1.27	ns
IL8 (pg/mL)	3.06 ± 1.53	2.47 ± 1.48	2.89 ± 1.50	3.94 ± 1.37	10.58 ± 7.85	6.79 ± 5.84	0.013
Candidate Gut-dysfunction Markers							
LPS ^c^ (EU/mL)	0.059 ± 0.019	0.093 ± 0.042	0.068 ± 0.029	0.040 ± 0.024	0.053 ± 0.011	0.046 ± 0.019	ns
LBP ^b^ (ng/mL)	1481 ± 1374	764.8 ± 808.9	1330 ± 1291	4290 ± 5906	7766 ± 1162	5780 ± 4620	*p* < 0.001
CD14 ^cd^ (×10^6^ pg/mL)	6728 ± 1681	9778 ±1153	7490 ± 2049	6350 ± 1111	8878 ± 780	7433 ± 1627	ns
Liver Cell Death Markers							
K18M65 ^ab^ (IU/L)	288.3 ± 92.2	205.7 ± 62.3	267.7 ± 91.8	2358 ±3098	864.3 ± 133.7	1718 ± 2333	0.008
K18M30 ^a^ (IU/L)	202.3 ± 48.4	300.9 ± 193.2	227.0 ± 107.3	386.6 ± 157.2	444.0 ± 81.3	415.3 ± 116.3	0.001
M65:M30^abc^	1.45 ± 0.44	0.80 ± 0.29	1.29 ± 0.50	2.04 ± 0.30	1.97 ± 0.33	2.01 ± 0.29	0.003

Significant between group analyses for: ^a^ males only between Gr.1 and Gr.2, ^b^ females only between Gr.1 and Gr.2, ^c^ between genders in Gr.1 only, and ^d^ between genders in Gr.2 only. ALT: serum alanine aminotransferase, AST: serum aspartate aminotransferase, AST:ALT: ratio of AST by ALT, IL-1β: interleukin 1 beta, IL-6: interleukin 6, TNF-α: tumor necrosis factor alpha, LPS: lipopolysaccharide, LBP: LPS-binding protein, sCD14: soluble cell of differentiation type 14, K18M65: soluble K18, K18M30: caspase-cleaved fragment of K18, M65:M30: ratio of K18M65 by K18M30.

**Table 4:** Liver injury measures, nutritional status, candidate blood panel measures, cytokines, gut dysfunction markers, and cell death markers of the alcohol use disorder patients after 2 weeks of SOC comparing patients by baseline FIB-4, tabulation by moderate (Gr.2c) and severe elevation of FIB-4 (Gr.2d).

Measures	Group 2c (1.45 ≤ FIB-4 ≤ 3.25), Gr. 2c)			Group 2d (FIB-4 > 3.25, Gr. 2d)			Between Group *p*-Value
	Males (*n* = 10; 71.4%)	Females (*n* = 4; 28.6%)	Total (*n* = 14; 51.9%)	Males (*n* = 9; 69.2%)	Females (*n* = 4; 30.7%)	Total (*n* = 13; 48.1%)	
Liver Injury Markers							
ALT(IU/L)	68.40 ± 21.70	27.00 *	61.50 ± 25.74	45.50 ± 19.02	63.00 *	49.00 ± 18.23	ns
AST (IU/L)	29.60 ± 7.02	30.00 *	29.67 ± 6.28	37.25 ± 5.85	39.00 *	37.60 ± 5.13	ns
AST: ALT ^a^	0.449 ± 0.094	1.111 *	0.560 ± 0.283	0.904 ± 0.305	0.619 *	0.847 ± 0.293	ns
Candidate Cytokine Response							
IL1β ^acd^ (pg/mL)	0.521 ± 0.282	0.971 ± 1.184	0.659 ± 0.671	0.427 ± 0.140	1.612 ± 1.809	0.822 ± 1.116	ns
IL6 ^a^ (pg/mL)	2.380 ± 0.989	4.260 ± 2.333	2.958 ± 1.681	4.527 ± 3.144	3.591 ± 1.071	4.215 ± 2.611	ns
TNFα (pg/mL)	2.329 ± 0.685	1.715 ± 1.045	2.140 ± 0.820	2.245 ± 0.909	3.275 ± 0.779	2.589 ± 0.974	ns
IL8 ^cd^ (pg/mL)	3.132 ± 1.613	6.042 ± 8.021	4.027 ± 4.446	3.414 ± 1.463	4.975 ± 4.475	3.934 ± 2.723	ns
Candidate Gut-dysfunction Markers							
LPS ^d^ (EU/mL)	0.063 ± 0.020	0.072 ± 0.020	0.065 ± 0.020	0.047 ± 0.020	0.085 ± 0.054	0.058 ± 0.036	ns
LBP ^c^ (ng/mL)	1641 ± 1574	2692 ± 3691	1883 ± 2084	2552 ± 4027	4570 ± 4297	3173 ± 4046	0.045
CD14 ^bcd^ (×10^6^ pg/mL)	6455 ± 1666	9425 ± 1506	7304 ± 2094	6862 ± 1498	9457 ± 644	7661 ± 1776	ns
Liver Cell Death Markers							
K18M65 (IU/L)	984.3 ± 2104.5	375.2 ± 357.9	810.3 ± 1782.5	435.1 ± 381.0	530.2 ± 379.7	464.3 ± 367.3	ns
K18M30 ^c^ (IU/L)	213.9 ± 58.8	353.6 ± 208.0	256.9 ± 132.8	252.2 ± 128.9	355.5 ± 154.0	284.0 ± 139.5	ns
M65:M30	1.564 ± 0.401	1.142 ± 0.866	1.434 ± 0.580	1.541 ± 0.560	1.340 ± 0.507	1.479 ± 0.532	ns

Significant between group analyses for: ^a^ males only between Gr.1 and Gr.2, ^b^ females only between Gr.1 and Gr.2, ^c^ between genders in Gr.1 only, and ^d^ between genders in Gr.2 only. ALT: serum alanine aminotransferase, AST: serum aspartate aminotransferase, AST:ALT: ratio of AST by ALT, IL-1β: interleukin 1 beta, IL-6: interleukin 6, TNF-α: tumor necrosis factor alpha, LPS: lipopolysaccharide, LBP: LPS-binding protein, sCD14: soluble cell of differentiation type 14, K18M65: soluble K18, K18M30: caspase-cleaved fragment of K18, M65:M30: ratio of K18M65 by K18M30. * Only single data point available.

**Table 5:** Liver injury measures, nutritional status, candidate blood panel measures, cytokines, gut dysfunction markers, and cell death markers of the alcohol use disorder patients at baseline comparing patients with improved K18M65 < 500 IU/L (Gr.2a) and patients with elevated K18M65 ≥ 500 IU/L (Gr.2b) within elevated FIB-4 patients (Gr 2).

Measures	Group 2a (Improved K18M65, Gr. 2a)			Group 2b (Elevated K18M65, Gr. 2b)			Between Group *p*-Value
	Males (*n* = 15; 75%)	Females (*n* = 5; 25%)	Total (*n* = 20; 74.1%)	Males (*n* = 4; 57.1%)	Females (*n* = 3; 42.9%)	Total (*n* = 7; 25.9%)	
Age (years)	47.5 ± 7.2	42.9 ± 12.0	46.34 ± 8.51	48.91 ± 6.93	49.76 ± 14.98	49.27 ± 9.95	ns
BMI (kg/m^2^)	24.8 ± 3.2	25.8 ± 2.6	25.05 ± 2.99	25.35 ± 2.44	25.20 ± 6.00	25.29 ± 3.87	ns
Drinking History							
TD90	1059 ± 478	911.4 ± 695.7	1022 ± 523	1226 ± 549	1317 ± 271	1256 ± 444	ns
HDD90	71.87 ± 23.08	75.80 ± 19.49	72.85 ± 21.80	85.50 ± 4.65	86.50 ± 4.95	85.83 ± 4.26	ns
AvgDPD90	14.96 ± 5.66	11.10 ± 7.14	13.99 ± 6.11	13.68 ± 6.19	15.33 ± 4.01	14.23 ± 5.19	ns
NDD90	72.93 ± 21.68	78.20 ± 16.39	74.25 + 20.21	89.75 ± 0.50	86.50 ± 4.95	88.67 ± 2.80	ns
LTDH ^c^	23.13 ± 9.57	9.40 ± 7.54	19.70 + 10.80	15.75 ± 11.50	18.33 ± 6.66	16.86 ± 9.10	ns
Liver Injury Markers							
ALT(IU/L)	103.8 ± 37.9	113.4 ± 112.6	106.2 + 61.9	82.25 ± 38.20	106.0 ± 106.1	92.43 ± 68.13	ns
AST (IU/L)	146.6 ± 89.2	154.2 ± 123.4	148.5 + 95.3	117.0 ± 47.2	223.0 ± 165.5	162.4 ± 116.0	ns
AST: ALT	1.35 ± 0.45	1.53 ± 0.63	1.40 + 0.49	1.70 ± 0.93	2.43 ± 1.03	2.01 ± 0.97	0.037
Nutritional Status							
CONUT	1.27 ± 1.16	1.00 ± 0.71	1.20 + 1.06	2.00 ± 2.45	0.33 ± 0.58	1.29 ± 1.98	ns
Blood Cell Measures							
WBC ^c^ (K/uL)	4.72 + 1.34	6.65 ± 0.95	5.20 + 1.50	5.81 ± 2.26	7.61 ± 3.12	6.58 ± 2.59	ns
AMC (K/uL)	0.398 ± 0.141	0.503 + 0.098	0.424 + 0.137	0.630 ± 0.403	0.468 ± 0.043	0.560 ± 0.299	ns
ANC (K/uL)	2.82 + 1.14	4.02 + 1.04	3.12 ± 1.21	3.43 ± 1.65	4.36 ± 1.92	4.07 ± 2.30	ns
Candidate Cytokine Response							
IL1β (pg/mL)	0.565 ± 0.281	0.564 ± 0.759	0.565 ± 0.437	0.373 ± 0.333	0.406 ± 0.269	0.387 ± 0.283	ns
IL6 ^ac^ (pg/mL)	2.45 ± 1.07	5.31 + 2.73	3.24 + 2.07	5.55 ± 5.30	8.78 ± 6.59	6.93 ± 5.61	0.022
TNFα (pg/mL)	1.88 + 0.57	2.30 + 1.69	2.00 ± 0.97	1.98 ± 0.24	2.54 ± 1.29	2.22 ± 0.82	ns
IL8 ^a^ (pg/mL)	4.63 + 2.28	7.18 + 6.47	5.34 ± 3.86	8.55 ± 5.42	23.75 ± 30.54	15.07 ± 19.79	ns
Candidate Gut-dysfunction Markers							
LPS (EU/mL)	0.121 ± 0.067	0.134 ± 0.070	0.124 ± 0.066	0.089 ± 0.060	0.075 ± 0.022	0.083 ± 0.045	ns
LBP (ng/mL)	2174 ± 2915	3140 ± 5347	2389 ± 3423	2576 ± 2681	1308 ± 343	2023 ± 2023	ns
CD14 ^bc^ (×10^6^ pg/mL)	9434 ± 1811	11218 ± 552	9980 ± 1763	8312 ± 2759	9373 ± 867	8767 ± 2092	ns
Liver Cell Death Markers							
K18M65 (IU/L)	1118 ± 1131	913.7 ± 1208.8	1067 ± 1121	1105 ± 502	1250 ± 411	1167 ± 434	ns
K18M30 (IU/L)	423.8 ± 420.1	535.8 ± 438.9	451.8 ± 416.0	347.1 ± 162.7	480.9 ± 79.4	404.4 ± 143.0	ns
M65:M30	2.53 ± 1.42	1.49 ± 0.67	2.27 ± 1.34	3.50 ± 2.01	2.57 ± 0.47	3.10 ± 1.53	ns

Significant between group analyses for: ^a^ males only between Gr.1 and Gr.2, ^b^ females only between Gr.1 and Gr.2, ^c^ between genders in Gr.1 only. BMI: Body mass index; TD90: Total drinks past 90 days; HDD90: heavy drinking days past 90 days; AvgDPD90: Average drinks per drinking day past 90 days; NDD90: number of drinking days past 90 days; NNDD90: number of non-drinking days past 90 days, LTDH: lifetime drinking history (in years), ALT: serum alanine aminotransferase, AST: serum aspartate aminotransferase, AST:ALT: ratio of AST by ALT, CONUT: Controlling Nutritional Status Test (unit: numerical), WBC: white blood cell count, AMC: absolute monocyte count, ANC: absolute neutrophil count, IL-1β: interleukin 1 beta, IL-6: interleukin 6, TNF-α: tumor necrosis factor alpha, LPS: lipopolysaccharide, LBP: LPS-binding protein, sCD14: soluble cell of differentiation type 14, K18M65: soluble K18, K18M30: caspase-cleaved fragment of K18, M65:M30: ratio of K18M65 by K18M30.

## Data Availability

The datasets analyzed during the current study are available from the corresponding author on reasonable request. Email address for the readers to contact the author to obtain the data: v0vats01@louisville.edu.
